# Precision Emotion and Affective Context (PEAC) process model: a theoretical framework for creating the affective circumstances that promote engagement

**DOI:** 10.3389/fpsyg.2025.1554099

**Published:** 2025-08-22

**Authors:** Stephanie M. Carpenter, Nicole A. Roberts

**Affiliations:** ^1^College of Health Solutions, Arizona State University, Phoenix, AZ, United States; ^2^School of Social and Behavioral Sciences, Arizona State University, Phoenix, AZ, United States

**Keywords:** engagement, affect, emotion, behavior change, intervention

## Abstract

Behavior change interventions are key to improving health and well-being. Knowledge of appropriate health behaviors does not, however, translate into engaging in them. We propose the “Precision Emotion and Affective Context (PEAC) Process Model of Engagement for Behavior Change” to serve as a theoretical framework to advance our understanding of how affect drives engagement and subsequent behavior change. Our framework highlights the importance of accounting for affective circumstances when designing and implementing behavior change interventions. The model accounts for affect that is both incidental and integral to the intervention: ongoing affective states that may work for or against the likelihood of engaging with the intervention (incidental), and affective states that are elicited by the intervention and consequently shape behavior change (integral). We focus on the promise of moderate levels of arousal and positive affective states, along with the need for contextual congruence, social acceptability, and personally meaningful approaches, for enhancing the likelihood of engagement and positive behavior change in interventions. Finally, just-in-time intervention approaches are discussed as a means of delivering personalized, affectively informed interventions that leverage advances in digital health technologies to promote opportunities for real-time implementation. This theoretical framework thus serves as a guide for constructing or harnessing the affective circumstances necessary to promote sustained intervention engagement and behavior change.

## Introduction

The prevalence of illness and disease is on the rise in the United States. This pattern is due in part to a proliferation of health compromising behaviors and chronic states associated with a risk of greater inflammation and illness, such as physical inactivity or sedentary behaviors, chronic stress, substance use, obesity, hypertension, and other chronic mental and physical health conditions. Much extant and ongoing research aims to develop interventions or tools that promote positive behavior change and can address these health compromising factors. These include, for example, health and lifestyle interventions to improve sleep, diet and exercise habits, and psychological or emotion-based interventions that teach cognitive restructuring, mindfulness, or other skills to improve mood, distress tolerance, perspective-taking, empathy, and future-oriented thinking (Gerber et al., 2023; Owen et al., 2018; Rizvi et al., 2011; U.S. Department of Veterans Affairs, 2024). Although such an approach holds strong promise in the ever-growing field of intervention science, even the most well-constructed interventions will only be effective at promoting behavior change if individuals use them. Indeed, engagement remains a critical challenge in behavior change research, as people often disengage from interventions and other behavior change approaches quickly and after minimal use (Nahum-Shani et al., 2022). In this paper, we outline a theoretical framework that highlights the importance of affective states as a way of promoting and sustaining engagement in behavior change interventions.

Engagement has been defined in many ways throughout the literature. Leveraging a recent framework for digital engagement (Nahum-Shani et al., 2022), we define engagement as a multifaceted construct that involves the investment of affective, cognitive, and physical energies (e.g., emotions, attention, information processing, actions) directed toward a specific stimulus (e.g., a message prompt encouraging a person to go for a walk) or task (e.g., the act of going for a walk). Engagement can be distinguished from adherence, which is conceptualized as participants following specific instructions to complete a task without the need for additional investment. In other words, adherence is necessary, but not sufficient, for engagement to be sustained over time. Research on intervention engagement increasingly has focused on ways to personalize interventions. Personalization involves incorporating information on an individual’s internal and external states to provide the right type of intervention support, at the right time (Carpenter et al., 2020). Whether or not a person chooses to engage in a particular behavior depends not only on their circumstances or external context at any given moment in time, but also on their own “internal” context, such as their emotional or affective state (Carpenter et al., 2016, 2020, 2023a, 2023b; Carpenter and Niedenthal, 2018).

“Precision medicine” and “precision health” are growing areas of interest in medical and public health spaces that seek to identify prevention tools and treatments personalized or tailored for each individual (e.g., based on their unique needs and interests) (Ashley, 2016; Hekler et al., 2020). We propose an analogous “precision emotion and affective context” framework (described in detail below) for optimizing the emotional/affective circumstances under which interventions, broadly defined, may be adopted to promote behavior change. In this context, the term “precision” does not require exactness, but rather acknowledges the window around an affective state that brings a relatively greater degree of precision than if it were considered more generally or not at all. Similarly, in medicine, there is not necessarily a perfect one to one mapping of a person’s needs or profile and their treatment, but considering the fact that different treatments may work more or less effectively for a given person can bring about relatively more precise treatment. We therefore use the term “precision emotion and affective context” as aspirational for guiding the field toward establishing an improved understanding of how interventions can leverage more of these person- and context-specific complexities when considering how best to promote behavior change. The proposed theoretical framework builds upon our extant understanding of engagement to provide novel and testable predictions for how to both promote and sustain *engagement* in behavior change interventions.

Although affective states are only one piece of the engagement process, we suggest here that identifying affective states and contexts that are particularly likely to promote engagement can increase the likelihood that people will use behavior change tools. This use, in turn, will increase the likelihood of lasting behavior change. In other words, identifying the affective circumstances under which to intervene can guide the development and implementation of interventions that are personalized to the individual and context to maximize engagement and behavior change over time. We suggest that interventions must both (i) generate and support emotional states that are most promising for promoting engagement and (ii) adapt to in-the-moment affective states that may disrupt engagement.

## Affective processes as guides to engagement

In this theoretical paper, we leverage our knowledge of affective processes and the way they can influence decision-making to make predictions about affective states under which engagement in a behavior change intervention may be particularly likely. We illustrate how interventions frequently administered through traditional (non-digital) methods can be designed or augmented to guide researchers and others delivering the intervention in understanding the affective circumstances that may promote or hinder engagement with respect to different intervention components. This information can help researchers and other interventionists (e.g., clinicians, providers, educators, community workers, etc. who deliver an intervention) shape which strategies, in what contexts, may be most useful for sustaining patient engagement and promoting meaningful behavior change. We also describe how digital interventions present an unprecedented opportunity to help assess or sense internal (e.g., affective, cognitive) and external (e.g., location) states and can adapt in real-time to deliver messages that either leverage or modulate affective states, as needed, to promote engagement.

*Affect* is an umbrella under which emotions, moods, and other experiences reflecting the physical state of the body (e.g., tired, on edge) may fall (Barrett, 2016; Gross, 1998). Affective states, including specific emotions, guide behavior in part by directing attention toward the elicitor of the emotion and mobilizing and prioritizing internal (and external) resources for action accordingly (Carpenter and Niedenthal, 2018, 2020; Ellsworth and Smith, 1988; Smith and Ellsworth, 1985). Furthermore, the experience of an emotion is largely shaped by several underlying situational appraisals. Such appraisals can include the perceived valence (positive or negative) of a situation, the intensity of the underlying arousal or activation associated with that situation, how in control (agentic) or certain an individual feels in that moment (Ellsworth and Smith, 1988; Smith and Ellsworth, 1985), and how the situation aligns with prior expectations (Puccetti et al., 2022), all of which can be shaped by cultural norms (Markus and Kitayama, 1991; Mesquita, 2022).

Appraisals therefore have implications for our perceptions and behavior. This includes, for instance, whether a situation is perceived as challenging and manageable, or stressful and a source of threat. Such appraisals have immediate and downstream implications, as cardiovascular and neural patterns corresponding to “challenge” rather than “threat” indicate that challenge experiences, as brought about by positive appraisals, are more sustainable and healthier longer-term (Blascovich and Mendes, 2000; Kampa, 2018; Seery, 2011; Tomaka et al., 1993; Wormwood et al., 2019a). Appraisals also shape tendencies toward approach or avoidance/inhibition and associated behaviors (e.g., as observed in resting-state neurophysiology) (Brosch and Sander, 2013; Deci and Ryan, 1985; Elliot, 1999; Sutton and Davidson, 1997). In addition to reflexive tendencies to approach safety and avoid harm, more complex motivations can shape a course of action. Different aspects of a situation, or competing goals and motivations within the same situation (Rusconi et al., 2022), can evoke both challenge and threat appraisals (Uphill et al., 2019). For instance, public speaking may be a stressful or physiologically “threatening” task that someone prefers to avoid, yet they would likely choose to engage in the task if it was required for their job. Similarly, social pressure to “have another drink” prior to driving home could result in pursuing the behavior (additional alcohol consumption) even if it overrides intrinsic safety or survival motivations. Overall, however, an individual is more likely to persist in achieving a goal when experiencing greater self-efficacy (Bandura, 2007), and/or external encouragement than when anticipating failure and feeling helpless or hopeless (Hadley and MacLeod, 2010; Maier and Seligman, 2016; Marchetti et al., 2023; Martinie et al., 2024). Similarly, even when motivated to pursue hard work to achieve a goal, tasks that are perceived as reasonably achievable engender more persistence than ones that do not (Hadley and MacLeod, 2010; Tice et al., 2007). Thus, appraisals are an important means through which affective states are shaped and in turn may link to behavior.

Affect and emotion also are intertwined with their regulation (Kappas, 2011), and both need to be accounted for when understanding what motivates human behavior. Implicit and explicit efforts to regulate current and anticipated emotions can exert influence on our behavior and decisions, such as via avoiding situations that are likely to generate unwanted emotions (“situation selection”; Gross, 1998, 2015), and even avoidance of the emotional experience itself (“experiential avoidance”; Gámez, et al., 2014; Hayes et al., 1996). Emotion and even the possibility of experiencing an emotion, and the strategies for amplifying, minimizing, or otherwise coping with these feelings, form the basis not only for action (Ekman, 1992; Frijda, 1987; Gross and Levenson, 1993; Majid, 2022), but also for inaction or avoidance (Hofmann and Hay, 2018; Kanter et al., 2008).

As discussed below, it is important for interventions targeting positive behavior change to allow for emotionally-congruent prioritization of the intervention; otherwise, paradoxically, stress can be induced from an intervention aiming to mitigate it. In today’s fast-paced, digital information-driven world where people are inundated with stimuli and need to choose where to focus their attention and efforts, it is especially challenging to make decisions aligned with favorable actions that will have benefits both in the moment and longer-term. Further, burden must always be considered when designing interventions. It is key to utilize information about an individual’s schedule and location to detect when they are likely to become overwhelmed, such as at certain times of the day or week, based on seasonal work cycles (e.g., midterm and final exam periods for students, tax season for accountants), family routines, or times when the person is simply unavailable (e.g., driving their car). This information can help tailor the timing of intervention delivery to better suit the individual’s needs and ensure that the intervention itself is not contributing to unnecessary burden.

Thus, engagement requires people to prioritize intervention-related components, such as recommendations, content, and tasks among other information and activities. This requires the individual to care enough about the intervention to prioritize it above other competing interests. One promising way to encourage such prioritization is to create an emotional circumstance that increases the appeal of engaging with the intervention. We present a model below that incorporates intersections of valence, arousal, and context as a framework for amplifying the role of affective context on engagement, with an emphasis on congruence between the affect an intervention elicits (integral) and the person’s ongoing affective state (incidental).

### Tipping the balance toward positive valence

Interventions can be designed to help promote positive emotional states, such as joy or interest, that are associated with approach motivations and encourage people to move toward (i.e., engage with) a stimulus or task (e.g., paying attention to a prompt that encourages a person to go for a walk; going for the walk) (Carpenter et al., 2013, 2023b; Fredrickson, 2001). Positive emotion also promotes persistence on difficult tasks (Carpenter et al., 2013; DeSteno et al., 2014; Tice et al., 2007). Even under conditions of sleep deprivation, which yields a powerful biological propensity toward a course of action (i.e., sleeping), positive emotion can largely override the impetus and result in sustained wakefulness and, in some cases, better cognitive performance when motivation is high (Alhola and Polo-Kantola, 2007). In other words, known, neurally instantiated links between positive affect and approach motivation (Davidson, 2004; Sutton and Davidson, 1997) [see also Harmon-Jones and Allen (1998) with respect to anger, as discussed below] underscore the relevance of positive emotion when designing interventions that will promote and sustain engagement.

Conversely, certain negatively-valanced states, such as fear, anxiety, stress, disgust, or sadness, may, in general, lead people to avoid a stimulus or task, and thus to disengage from it (Ekman, 1992; Frijda, 1987; Hofmann and Hay, 2018; Kanter et al., 2008). Such negative emotions may be perceived as more effortful to experience and endure, especially among those who already experience clinical symptoms (Gámez, et al., 2014; Roberts et al., 2020). Evidence suggests that such states can be motivational in the short-term, such as fear of needing to take insulin resulting in reducing sugar intake, or fear of losing a promotion resulting in working through weekends and holidays. However, this kind of motivation is likely to be challenging to sustain in the long run and can even lead to burnout and disengagement (Maslach and Jackson, 1981; Maslach and Leiter, 2016). Rather, engagement as defined here is a process that goes beyond a person aiming to overcome negative consequences and instead involves embracing a new approach to achieve positive behavior change (consistent with the idea of creating an “upward spiral” of positive affect and behavior; van Cappellen et al., 2018). Nevertheless, positive and negative states often co-occur (Vaccaro et al., 2020), and there are circumstances under which engagement can still occur or even be accelerated amidst negative states. This may be especially likely to occur if an intervention is perceived as worthwhile, therapeutic, or otherwise supportive of the regulation of experienced negative emotions. For instance, sadness may drive engagement if an intervention is perceived as one that can help the person overcome or reduce their feelings of sadness (e.g., through journaling or activity scheduling; Dimidjian et al., 2006; Dinç et al., 2024; Pantchenko et al., 2003). Similarly, approach motivation associated with anger (Harmon-Jones and Allen, 1998) can be leveraged to encourage constructive behavior such as exercise or leaving a high-conflict relationship. While anger in and of itself can be compelling when the outcome of the anger is likely to have rewarding or otherwise positive consequences (Lench et al., 2024) (e.g., anger at a tennis opponent motivating harder hits and winning the game), in many contexts anger also can encourage people to take risks that result in problematic consequences (e.g., anger leading to “road rage” while driving may cause an accident; Kjaervik and Bushman, 2024). Overall, we suggest that the intervention itself should engender a sense of pleasantness and perceived reward directly, and/or be paired with such states to facilitate engagement indirectly.

Thus, highly engaging interventions should promote generally positive affective states, through both a primarily positive tone (Carpenter et al., 2023a) and intervention content that will help people regulate and redirect the experience of negative emotions.

### Moderate physiological arousal can promote engagement

The intensity of any experienced affective states is also key to the engagement process. A physiological arousal state that is either too high or too low may hinder engagement, because the participant is distracted by the physiological state and/or is not motivated to engage with something that could disrupt this state, especially if it is experienced as desirable.

For instance, imagine a positive emotion state where physiological arousal is very high – such as in the case of elation (e.g., having just received exciting news). Here, the high arousal physiological state may distract individuals from noticing an intervention prompt, especially if the prompt is unrelated to the source of the elation, or may lead the individual to ignore the intervention even if they technically noticed the prompt. Similarly, if the physiological state is quite low, such as when experiencing a calm or relaxed state (e.g., lying on the couch reading), an individual may not be motivated to respond to a message prompt or engage in a task (going for a walk) that might alter the physiological state.

As such, we propose that an ideal physiological state for engagement would follow the well-known “inverted-U” curve, whereby performance is maximized with moderate levels of psychological or physical stress or arousal (Arent and Landers, 2003; Diamond, 2005; Yerkes and Dodson, 1908). We posit this while recognizing the influence of contextual and other intervening factors (discussed below) that may alter the nature of arousal and brain-behavior relationships and result in different patterns of non-linearity with respect to performance and motivation (Diamond, 2005). Thus, in general, moderate levels of arousal are predicted to motivate greater intervention engagement than an extreme state marked by either very low or very high physiological arousal.

We note that physiological arousal could be measured objectively with devices that measure physiology, or subjectively based on individual self-report. These two metrics may or may not be related, and their degree of association can vary between different individuals and at different times within the same individual, but they nevertheless each are a source of information. For example, when using a wearable device, an “objective” heart rate increase could trigger a digital intervention prompt, but someone could also track their subjective experience and be trained to utilize intervention strategies (e.g., listen to a meditation audio clip) based on self-ratings. Someone also could be guided to infer their state from a behavior, such as if they find themselves quickly pacing back and forth, or have a difficult time mobilizing to do a needed chore. In other words, depending on the intervention modality, study design, and/or available resources, “arousal” can be conceptualized and measured in several ways, all of which could be harnessed to help guide and create the affective circumstances that promote engagement.

### Emotional circumstances underlying engagement: considering integral and incidental affect

The discussion thus far is based on the general notion that engagement involves understanding both *integral* and *incidental* affect. Integral affect is evoked by the intervention itself, whereas incidental affect reflects ongoing affect that is elicited by the context and circumstances (Isen and Daubman, 1984; Schwarz and Clore, 1983). Interventions can be designed to help people directly modulate emotional states they bring to the intervention that may be particularly disruptive to the engagement process, such as fear or stress (incidental affect). In addition, interventions must be developed to elicit generally positive emotions, such as interest, excitement, happiness, or gratitude (integral affect).

**Figure 1:**
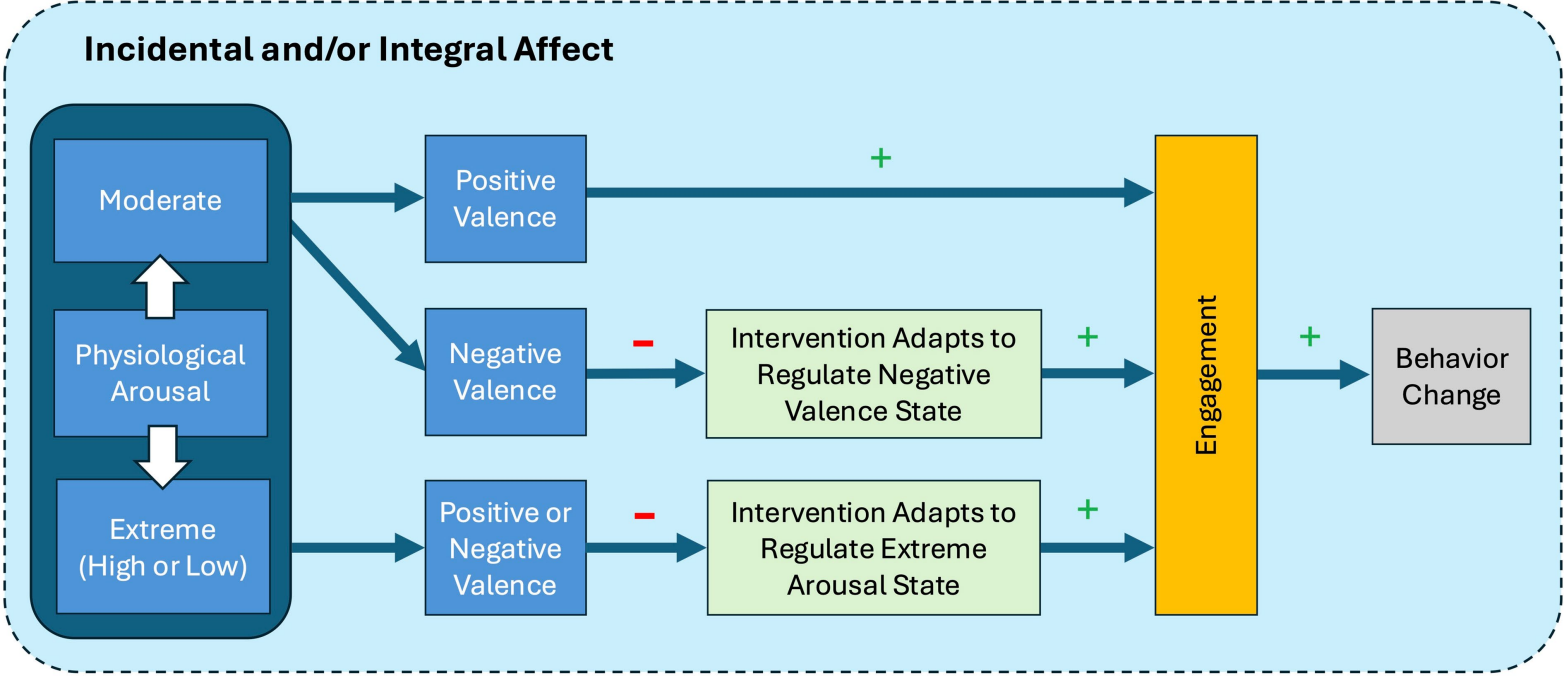
Precision Emotion and Affective Context (PEAC) Process Model of Engagement for Behavior Change. (+) denotes a predicted increase, and (−) denotes a predicted decrease. This figure depicts a testable path through which we predict that moderate levels of arousal and positive affect will yield a state where individuals are most receptive to an intervention, and thus most likely to engage, leading to subsequent behavior change. Further, this model predicts that interventions that adapt to accommodate extreme arousal states (irrespective of valence) or negative states will also promote engagement. Although physiological arousal is depicted first (leftmost) in the model, this is not intended to imply that arousal must precede valence perceptions/judgments; rather, these may occur simultaneously, sequentially in either direction, or independently. Incidental affect refers to the ongoing ambient (mood or context-based) affect, and integral affect is generated (purposefully or not) by the intervention itself.

Here, we propose the term “Precision Emotion and Affective Context” to refer to the process by which researchers can harness and create emotional circumstances to promote greater engagement in positive behavior change. This precision emotion process involves both the elicitation of affective states that promote engagement, and the regulation of affective states that hinder engagement. As described above and in Figure 1, the precision emotion process involves an understanding of how both valence and arousal impact engagement. Through dynamically shaping one’s affective environment, we propose that the theoretical *Precision Emotion and Affective Context (PEAC) Process Model of Engagement for Behavior Change* can guide researchers in incorporating emotions and regulatory strategies that more effectively elicit and sustain engagement in their behavior change interventions. This framework can be applied to both every day and more consequential decisions that a person must make that can either benefit or be detrimental to their health and well-being. For instance, people with health conditions routinely enroll in interventions and research studies, particularly clinical trials, to achieve a benefit for themselves or others (Johns Hopkins Medicine, n.d.; National Institutes of Health, 2019). Given that affective circumstances shape decisions (Carpenter et al., 2013, 2016, 2023a; Carpenter and Niedenthal, 2018; Nahum-Shani et al., 2022), researchers should consider the implications of both the affective states that people bring to the intervention, as well as how participation in the intervention shapes concurrent and subsequent affective experience. It is also important to consider that people can value and experience affective states differently (Tsai et al., 2006). For example, “pride” may be considered positively valenced and motivating in one culture, and something to hide and avoid in another (Kim-Prieto and Eid, 2004; Kitayama et al., 2000). This nuance is accounted for in the PEAC process model when harnessing the affective circumstances necessary to promote intervention engagement and behavior change.

### Creating personally meaningful interventions

Meaning making and personal relevance are key motivators (Bonanno, 2013; Carpenter et al., 2023a, 2023b; Lewis, 2020). Such motivators are often tied closely to social relationships, because people derive meaning, motivation, and strong emotion from interactions with others (Hawkley et al., 2005). Thus, to achieve emotional circumstances that promote engagement, it is essential to link the intervention in meaningful ways to a person’s self, identity, and/or social motivations, in addition to their stated goals.

Further, it is plausible that people find self- or identity-relevant information more emotionally salient, from both a physiological arousal and a valence perspective (Carpenter et al., 2023b; Roberts and Levenson, 2006). Accordingly, self- or identity-relevant information is predicted to be especially likely to elicit affective states that grab attention and guide action. By creating interventions that are congruent with someone’s view of their current or ideal self it should reduce cognitive dissonance and promote engagement (Carpenter et al., 2016, 2023b; Festinger, 1962). For example, someone who views themselves as extraverted would be more likely to participate in a group-based intervention than someone who identifies as an introvert; someone who avoids technology would be less inclined to use it than someone who views themselves as an “early adopter” of the latest tools (Anderberg et al., 2019). Self- and socially-relevant identity also extends to racial or ethnocultural background, gender, religion, or other kinds of identity that may be personally meaningful. Social identity or group membership may be especially important for those from backgrounds that value a more “interdependent” orientation (Cross et al., 2000; Kitayama et al., 2022). For example, a strong sense of *familism* in Hispanic/Latine cultures may require aspects of an intervention that can garner the support of family members, or integrate with and perhaps even benefit family obligations (Marin and Marin, 1991; Santiago-Rivera, 2003). This circumvents the psychological conflict and negative affect that can occur (and in turn compromise intervention engagement) if an intervention advances some goals (e.g., exercising) at the expense of others (e.g., family time). As another example, if someone engages in regular religious practice at certain times each week, an intervention would ideally be respectful not to disturb this schedule, and instead to build on it where appropriate. Thus, to construct an intervention that adequately generates the intended emotional circumstance, it is important that the intervention is viewed as relatable, or elicits the feeling that it was designed with the person in mind (Carpenter et al., 2020, 2023a, 2023b).

Social motivation is a particularly powerful driver of behavior (Maslach and Jackson, 1981). An optimal intervention therefore may integrate support from a coach, peer, or close other (e.g., family member, friend). In an ideal scenario, an intervention could be one that close others can support and potentially share in, or, at minimum will not discourage the person from pursuing. Smartwatches, standing desks, yoga classes, and fitness trackers are examples of health-related intervention components that have become normative in many social circles and contexts, including the workplace (Buman et al., 2017; Lewis et al., 2017). At the same time, many health-related interventions are stigmatizing, such as ones that address obesity, sexually transmitted infections including HIV, or addiction. People may want to conceal their participation in such interventions from others. In these cases, a support system could be built into an app via coaches or peers with a similar condition or goal. Such approaches also would be applicable to those experiencing social isolation (with or without a stigmatizing health condition) who may not have a network to rely on for support. Thus, developing interventions that support socioculturally-congruent motivations and contextual understandings are likely to be appealing and thus promote engagement in the intervention.

## Precision Emotion and Affective Context (PEAC) Process Model of Engagement for Behavior Change

Figure 1 presents a process model capturing our proposed theoretical framework for affectively driven engagement and subsequent behavior change. Specifically, we introduce the Precision Emotion and Affective Context (PEAC) Process Model of Engagement for Behavior Change, which guides the construction of affective conditions that promote engagement in positive behavior change. Importantly, we note that while the model is linear in its presentation, affective components (arousal, valence) are mutually influential and recursive. This process model is couched in both the incidental affect that participants bring with them to an intervention context, as well as the integral affect that is created by the intervention itself.

First, to create an ideal emotional circumstance wherein engagement in key aspects of the positive behavior change intervention is likely, we start with consideration of one’s physiological arousal state (left side of Figure 1). As noted above, this can be assessed “objectively” via a wearable sensor, as well as subjectively via self-report [with an understanding that these may or may not correlate, and that there are individual differences in arousal-appraisal and arousal-experience links (Hoemann et al., 2020; Wormwood et al., 2019b)]. Context-based assessments also contribute information about arousal (Hoemann et al., 2020) and may require less user input (e.g., during a regularly scheduled exercise class, or a typically stressful weekly work meeting or presentation).

It is expected that, in most cases, there will be an interaction between one’s degree of physiological arousal and valence in predicting behavior (left-middle of Figure 1). For example, people experiencing moderate physiological arousal while in a positively valenced affective state (e.g., joy, hope) will be more likely to engage with a stimulus (e.g., reading an intervention prompt sent to one’s cell phone encouraging them to call a friend) or a task (e.g., calling the friend).

Conversely, people experiencing moderate physiological arousal while in a negatively valenced affective state (e.g., fear, anxiety, sadness) will plausibly be less likely to engage with an “irrelevant” stimulus or task (e.g., a prompt for a stress-reduction intervention). However, if the intervention can adapt based on sensing internal and/or external contexts to regulate the negative affective state (middle-right of Figure 1), then it will not only increase the likelihood that a person engages with a given stimulus or task, but will also have a mood regulation benefit.

In such a scenario, for example, a smartphone app presenting a menu of affective states (e.g., asking which you are feeling right now) with corresponding options (e.g., if sad, call a friend; if stressed, go for a walk) could be presented. Over time and with repeated exposure to options for constructive coping or recommendations for other positive behaviors, individual preferences can be learned (e.g., through digitally delivered prompts, guidance from the research team or other interventionists, or even oneself). Thus, in subsequent iterations, intervention options can be presented based on someone’s preferred actions when in prior similar states (e.g., when disappointed, instead of consuming sweets or vaping, one person prefers to listen to music, whereas another prefers to engage in an activity at their community center). In other words, interventions can be personalized for real-time delivery, and people’s preferences for what intervention options to engage with also will be reinforced by repeated instances of their own behavior (Robles et al., 2011). This is important, as it is much easier to leverage existing preferences than to change them.

We propose that people experiencing more extreme (high or low) physiological arousal regardless of affective state will plausibly be less likely to engage with an intervention-related component, such as a recommendation or task. This is because internal resources necessary for engagement will presumably be directed toward the source of the high arousal state. Conversely, in the case of extreme low arousal states (e.g., sleep, watching a low arousal movie or TV show while seated on a couch) the individual will need too much stimulation to mobilize. However, if the intervention includes technologies (e.g., wearables) that can sense the extreme arousal (high/low) state and adapt to deliver an appropriate self- or identity-relevant message that aids in the regulation of this arousal state, then this will plausibly increase the likelihood of engagement. Even when interventions do not integrate digital technologies, a person could be instructed by a researcher or other interventionist to attend to specific cues (e.g., feeling irritable or on edge) as a signal to self-regulate (e.g., identifying overwhelming tasks, engaging in positive self-talk) to then be able to engage in a primary intervention-related behavior (e.g., good nutritional choices; social engagement for someone who feels lonely). In this sense, engagement is expected to promote positive behavior change, which is the desired outcome of many interventions. Importantly, the proposed PEAC process model can guide both initiating and sustaining engagement over time to establish habits that result in meaningful and lasting behavior change. We also do not view specific emotions and arousal states as absolute; although valence and arousal are key components of human experience, their interpretation undoubtedly varies widely, especially when greater specificity is introduced (e.g., specific emotions).

## The PEAC process model in action

We will now illustrate the proposed PEAC model depicted in Figure 1 by providing predictions for ways in which personalized self-management interventions designed to promote real-time, real-world positive behavior change can create emotional circumstances that promote engagement. We provide examples of three different positive behavior change interventions that each have implications for health and well-being: one seeking to increase daily physical activity, one seeking to curb rumination in the context of social anxiety, and one targeting the management of high and fluctuating levels of job stress experienced by law enforcement officers.

### Everyday engagement in physical activity

Imagine that an individual is enrolled in a digital health intervention that aims to encourage greater physical activity to reduce the likelihood of illness and disease, such as diabetes, heart disease, or chronic pain. In this intervention, engagement in physical activity is prompted via message prompts (texts) sent to one’s smartphone that encourage different sessions of walking. In a moderately aroused, positive affective state, the proposed PEAC framework (see Figure 1) predicts that the individual will feel motivated toward action; this motivation, in turn, will make them feel capable of going for the walk. In this positive state, they would be more likely to notice the prompt in the first place and feel more positive carrying out the act of going for the walk. Conversely, if the individual is in a tense, stressed state, it may be a time when the walk is most needed, yet the prompt is most likely to be ignored.

If in an extreme arousal state, we predict that the individual will plausibly feel less motivated to act because they are distracted away from the intervention (e.g., they do not notice the prompt) or feel too overwhelmed to either read the prompt or go for the walk. In the case of a low arousal state, they may not feel sufficiently motivated to read the prompt or may not have the energy to go for the walk. This lack of energy directly translates into a lower motivation to carry out the act of walking.

One might wonder how an intervention can adapt to modulate either negative affect or extreme physiological arousal. On a day when an individual may be likely to feel negative emotions, such as when they have an upcoming dental procedure (e.g., as identified on a synced calendar), the intervention can deliver a message prompt that helps the participant reduce negative emotions they may be feeling, such as reminding them of how taking even a brief walk can help reduce stress or fear associated with the upcoming appointment. Such technologies are often already integrated into people’s mobile devices (e.g., a GPS location tracker on a smartphone) and can be seamlessly employed to ensure the right type of intervention is being delivered when an individual needs it most.

For instance, many wearable devices, such as activity trackers, are also capable of sensing physiological arousal (Can et al., 2019). If one such device detects an extremely high physiological state, and it can be inferred from the person’s location or the time of day that they are not currently exercising, then an intervention prompt encouraging a self-regulation activity that reduces physiological arousal (such as deep breathing exercises) can be delivered prior to any instruction to engage in the target physical activity.

Finally, if determining a friend is currently walking, or will be soon, that information could be used to prompt the person to walk accordingly. However, if there was a recent fight with the friend, timing an intervention to walk with them would be contraindicated. This example illustrates how quickly and unpredictably affective-relevant contexts can change, and suggests that frequent sensing and assessment of contextual factors is warranted to ensure adequate information is being collected for adaptation over time.

### Promoting engagement in a cognitive behavioral therapy (CBT) intervention

Imagine that a clinical psychologist delivers an evidence-based CBT intervention to help a client manage rumination in the context of social anxiety. During weekly counseling sessions, the clinician provides CBT training to the client and instructs them to complete a daily diary to indicate the feelings they experienced throughout the day, particularly before, during, and after social situations, and practice CBT techniques (e.g., writing down negative thought patterns that can be challenged and replaced with more realistic ones via cognitive restructuring; evaluating evidence to challenge beliefs about what others may be thinking). During the weekly appointment, the clinician likely would work with the client to understand the barriers to implementing the CBT exercises, which could be affectively-related, and to understand what prevented them from not fully completing the thought record, evidence evaluation log, or other homework/practiced techniques.

Following the PEAC model, the clinician would continue to build on this approach by being intentional about the affective states – both related to the presenting complaint and more broadly – that are likely to facilitate versus deter the client from adequately engaging in the CBT intervention. The clinician can then guide the client in identifying affective states (e.g., extremely low or high arousal, negative valence) that disrupt their engagement in the CBT intervention in a given moment or in the time frame leading up to the point of engaging or not engaging in the intervention (or doing so partially) and discussing strategies for regulating these feelings. For example, the client with social anxiety may be too fatigued or “frantic” from working on a challenging task (unrelated to the presenting problem), or from staying up late ruminating about attending a social gathering (related to the presenting problem), to complete a log with evidence supporting or refuting their negative thoughts about the upcoming social interaction. Addressing the fatigue or “frantic” feeling could translate to a greater ability to engage in the intervention. In the future, when the client experiences disruptive affective states (e.g., fatigued, frantic), the state itself can cue them to apply a regulatory strategy to reduce the disruptive affective state and promote engagement in CBT. This is not incompatible with current approaches to addressing “therapy-interfering behaviors” in dialectical behavior therapy and other treatments (Allen, 1997; Chapman and Rosenthal, 2016). The PEAC model aims to complement these approaches while both broadening to consider different kinds of emotions and affective circumstances in daily life, and applying them to treatments or interventions that are not already considering engagement as centrally. Placing more focus on creating the affective circumstances that promote engagement will plausibly bolster the effectiveness of evidence-based interventions like CBT.

### Police officer engagement in stress regulation

Law enforcement officers, such as police officers, are good candidates for stress regulation interventions because they often find themselves in highly stressful situations on the job (Can and Hendy, 2014; Verhage et al., 2018). These situations may punctuate moderate or underwhelmingly low levels of stress (under-arousal) or boredom (Phillips, 2016), providing an opportunity to track and intervene prior to the onset of a highly-arousing experience (Baldwin et al., 2019; Fridell and Binder, 1992).

Although police officers receive extensive training at police academies, many still benefit from practicing in-the-moment stress regulation skills (Andersen and Gustafsberg, 2016; Arnetz et al., 2017; Jetelina et al., 2020; Otte et al., 2005; Reingle Gonzalez et al., 2019; Sim et al., 2022). Such skills are needed to reduce the likelihood of impulsive actions that may accelerate a conflict and lead to greater violence, placing both the police officer and community members at risk (Andersen et al., 2020; Violanti, 2007). Further, police training paradoxically may be at odds with stress regulation, in that officers must learn out of necessity to be in a high-alert state much of the time (Arnetz et al., 2013; Roberts and Levenson, 2001) and to suppress (versus acknowledge) their anger (Can and Hendy, 2014). Thus, they are a good use case for considering our PEAC framework.

As is the case with many occupations, job-related chronic stress is also a major factor linked to the prevalence of poor health coping behaviors among some police officers, such as the use and misuse of alcohol and other substances (Beehr et al., 1995; Can and Hendy, 2014; Lindsay, 2008). This, in turn, has several longer-term health consequences, including increased rates of hypertension, heart disease, diabetes, obesity, and other illness.

Consequently, for a stress-regulation intervention to appeal to police officers, it must take into consideration the regulatory activities that are feasible for a police officer to carry out, as well as leverage the police officer role identity (i.e., psychological identification as a police officer) in ways that feel meaningful enough to promote connection to and prioritization of the intervention.

Imagine a police officer is enrolled in a digital health intervention to encourage engagement in stress reduction activities to help reduce stress and stress-related consequences (e.g., impulsive actions, sleep disruption, long-term disease) – which in themselves become sources of physical and psychological stress. Stress reduction activities are delivered in this intervention via message prompts (texts) sent to one’s smartphone or smartwatch that encourage engagement in different brief self-regulation activities. Based on our PEAC framework, we predict that in a moderately aroused state, an officer will be able to think clearly enough to pause and implement a practiced, ideographically preferred technique (Jetelina et al., 2020), such as paced breathing, envisioning a loved one, viewing a photograph of their pet, listening to a favorite song, stepping away for a “time out,” or remembering their goals and values. Further, we predict that each of these different techniques may not be equivalent in terms of the degree of cognitive resources and energy required for an individual to successfully carry them out, which may further hinder an individual’s ability to implement them in a real-time, real-world situation (Bonanno and Burton, 2013).

In such cases, it is not just the arousal state, but also the context, that is critically important. Even if a moderately aroused state might typically be conducive to engaging in an intervention, doing so may not be feasible under very constrained circumstances. For instance, an officer trying to calm down someone in a crisis may not be able to spare a moment to reduce their own stress by taking out their phone and viewing a joyful photo. Creative, context-congruent solutions for intervention delivery are often needed, such as sending a vibration that the individual learns to associate with adjusting their breathing or reconstruing an event to align with a (previously-practiced) thought.

Similarly, we predict that in an extreme high arousal state, a prompt serving as a reminder to “shift gears” may be effective (although arguably less so than with moderate arousal) at points in time when an individual has a moment to engage with the prompt. Such moments may occur at the beginning or end of a workday or in-between calls. During moments of active on-duty focus for a law enforcement officer, any prompt could be disruptive and even dangerous. Thus, understanding the affective circumstances leading up to critical moments may be particularly important to delivering interventions when a person is available, as well as receptive (willing and able), to receive the prompt.

A low arousal state (e.g., signaled by relatively lower heart rate and low reports of stress) (Sim et al., 2022), as may be more likely to occur during slow (e.g., while handling paperwork) or off-duty hours, is predicted by our PEAC framework to require a motivational “pre-” intervention. For instance, at the end of a shift prior to the drive home, a reminder about the benefits of taking time to prepare a healthier meal versus buying fast food, or the benefits of getting extra sleep instead of staying awake surfing the internet, could be delivered outside of (in this case, before) the scheduled time frame of the intervention. As noted with respect to high-arousal states, the intervention could be cued or signaled with a minimally intrusive approach, such as a catch-phrase, tone, vibration, or image, in order to avoid annoying or over-stimulating the individual.

Waiting for arousal levels to shift naturally, rather than “forcing” an intervention at an inappropriate time when the intervention will be perceived as an annoyance or burden, is likely necessary to maintain engagement. As an alternative or additional approach, however, having an authority figure (e.g., department chief) endorse the use of an intervention across a cohort may bolster engagement when an individual’s personal motivation is low. Such an approach that relies on other-driven/extrinsic motivation and reduces the need for self-driven/intrinsic motivation may be best for interventions that are needed only in the short term (e.g., in response to a unique or time-limited circumstance). Such approaches may be less well suited when the goal is to establish longer-term habits and lasting behavior change.

## Further applications of the PEAC process model of engagement

### Minimizing particularly unpleasant self-conscious emotional states

In addition to those described above, there are several self-focused and social–emotional states or contexts that are likely to further inform our understanding and application of the proposed PEAC process model. Here we focus on two: shame and embarrassment.

Of all emotions, shame arguably is most closely linked with avoidance motivations. Shame is a culturally and socially-focused emotion that leads the individual to feel in some way deficient. Notably, its definition includes the experience of wanting to hide (Stuewig et al., 2015; Tangney et al., 1992). Thus, interventions that are shame-inducing in any way are particularly unlikely to gain traction or promote engagement and may dissuade a person from participating in any future interventions.

While shame has several distinct features (e.g., turning down of the head; Tracy et al., 2009) and neurobiological correlates (e.g., activation of neural regions involved in self-referencing; Zhu et al., 2019), it may be difficult to detect in real time without a specific, easily trackable physiological (autonomic) signature (Hoemann et al., 2020). However, it is possible to examine the mismatch between goals and actions (e.g., a fitness goal coupled with long sedentary periods; an anger management goal coupled with vocal outbursts), and design interventions that seek to reduce this mismatch. As noted earlier, many health conditions are stigmatizing, and therefore it can be particularly important to take a positive, future-oriented versus mistake-oriented approach.

In other words, part of designing an affectively sensitive intervention involves cultivating rewarding experiences versus producing (often unintended) punishment. Although in some ways this may seem obvious, it is not what many “motivational” interventions (e.g., delivered via smartphone apps) achieve in practice. Even in cultures where shame is more accepted and used as a motivational tool (e.g., in parenting), the bulk of the evidence to date suggests non-shame experiences may be more effective at promoting engagement (Li et al., 2004; Smetana et al., 2021).

Although perhaps less extreme or lasting than shame, feelings of embarrassment, which may co-occur with shame or on their own, are expected to also discourage engagement. Again, embarrassment may be evoked directly or indirectly by different aspects of an intervention (e.g., a loud noise from a prompt in an otherwise-quiet social situation; a notice from a research team member conveying a failure to meet a specified goal). Based on our PEAC framework, we predict that an intervention designed to reduce or avoid emotions such as shame and embarrassment, while also appealing to identity-congruent motivations, will plausibly have maximal benefit on sustained engagement and subsequent behavior change. As such, we predict that positive self-relevant emotions (e.g., pride), and other-focused emotions (e.g., gratitude) may also be beneficial to promoting intervention engagement.

### Just-in-time adaptive interventions

Just-in-time adaptive interventions (JITAIs) sense in-the-moment information about a person’s internal and external contexts and are designed to use this information to deliver the right intervention support, at the right time (Carpenter et al., 2020; Nahum-Shani et al., 2015, 2018). JITAIs often rely on digital technologies (e.g., wearable devices, smartphones) to aid in the sensing of real-time internal and external states that drive the adaptation process. This adaptation process, in turn, allows for the delivery of the right intervention in the moments when people are most able and willing to engage. Yet, many questions remain about under what conditions it is best to deliver these interventions, as well as how best to personalize an intervention to meet the needs of different groups of people, or even different individuals.

JITAIs hold promise in the promotion of precision emotion because they can adapt to provide the right support, at the right time, depending on a person’s affective and contextual state. Experimental designs, such as microrandomized trials (Carpenter et al., 2020; Klasnja et al., 2015; Qian et al., 2022), that aid in the development of efficacious JITAIs allow for the collection of real-time, real-world data on affective states and contextual factors to more precisely tailor interventions. Further, although not necessary for constructing JITAIs, artificial intelligence (AI) learning of state-action preferences can also be employed as part of JITAIs to predict similar future states and corresponding actions. In the context of the proposed PEAC model, AI tools, although still in their infancy, have the potential to identify and learn over time which components of an intervention an individual prefers (Arefeen et al., 2022; Nahum-Shani et al., 2024) when in different affective states to inform the intervention adaptation process. Below we provide an example of one affective context (i.e., affective forecasting) that may be particularly promising for a JITAI design.

### PEAC and JITAI implications for affective forecasting

Affective states by definition fluctuate, and people often incorrectly forecast their future affective states (Wilson and Gilbert, 2003). For instance, individuals in a physical activity intervention may incorrectly forecast that going to the gym today will make them feel more fatigued, or a law enforcement officer may incorrectly forecast that they will not feel stress during an arrest. Anticipating these affective forecasting errors before they occur can be critical to preventing emotion-linked moments of poor decision-making. Indeed, facilitating recognition and labeling of one’s own affective patterns can itself be a useful intervention (Hülsheger et al., 2013; Lieberman et al., 2011).

Starting out by implementing JITAIs that adapt to deliver regulatory strategies for practice during times when arousal is moderate and affect is positive, as per the PEAC framework, can pave the way for implementing these strategies at the times they are most needed. For example, mindfulness breathing works best if practiced daily, and then once the behavior has become practiced and habitual, it becomes more automatic and can be called upon during high stress situations (Bockmann and Yu, 2023; Kabat-Zinn, 2003). Such JITAIs may help reduce the extent to which people experience errors in their affective forecasts by providing the opportunity to both practice regulatory strategies when they have the resources available to do so, and better learn how to predict when a situation may be better or worse than anticipated. Further, improving day-to-day healthy behaviors, which is linked with improved resting-state or “baseline” physiology, not only is beneficial overall, but plausibly also facilitates faster recovery when a person goes off track from their goals (e.g., eating poorly when on a diet; lapsing in smoking or heavy alcohol use).

Self- and therapist-assisted monitoring and tracking has been the cornerstone of many psychological therapies for decades, including CBT for depression and anxiety (Beck et al., 1979); interpersonal and social rhythm therapy for bipolar disorder (Steardo et al., 2020); dialectical behavior therapy for borderline personality, suicidality, and emotion dysregulation more broadly (Linehan et al., 2006); and cognitive/neurobehavioral therapy for neurological symptoms and their functional counterparts (e.g., traumatic brain injury, epilepsy, and non-epileptic/functional seizures; LaFrance et al., 2009; Van Patten et al., 2024). These approaches have been translated into mobile interventions that provide recommendations and “coaching,” which can facilitate healthy coping behavior even in the absence of a therapist (Rizvi et al., 2011). Despite the complexity of human emotion and behavior, and the centrality of the patient-provider relationship in promoting positive behavior change, advances in machine learning and other types of AI show initial promise for pattern detection and prediction. This has the plausible benefit of requiring less reliance on user inputs and awareness in circumstances where these are not as easily obtained (e.g., when working with individuals who have low levels of self-awareness, or who are unable to report on their affective states), and improves adaptation and timing of intervention delivery. Notable examples of such real-time detection include blood glucose tracking for diabetes (Arefeen et al., 2022), predicting length of time in a sedentary posture (e.g., sitting) versus engaging in physical activity such as “steps” (Arefeen et al., 2022; Buman et al., 2017), and predicting psychologically-complex behaviors like online bullying among youth (Silva et al., 2016) or stress among workers (Martinez et al., 2022). These may augment, rather than replace, human involvement in the process.

It is important to note that while awareness of affective states enhances self-regulation and well-being, individual differences in this awareness, particularly experiences of physiological arousal, can hinder an individual’s ability to effectively use this information in real-time, real-world contexts. Further, although some digital interventions may aim to ease the burden of constant user inputs by minimizing self-monitoring of affective states and associated behaviors, real-time interventions also have the potential to *enhance* self-awareness. For instance, a real-time recommendation acknowledging that an individual may currently (or soon) be experiencing a disruptive negative affective state because of a stressful meeting and offering a real-time regulatory strategy can enhance the individual’s awareness of their current affective state and provide a tangible suggestion for how they can manage it before it disrupts engagement in behaviors of interest. Such interventions show promise for teaching individuals under what affective conditions it is beneficial to use different regulatory strategies and plausibly promote flexible regulatory strategy use even after the intervention has ended.

Additionally, some digital interventions are *designed* to enhance awareness of internal and external states through frequent self-monitoring via ecological momentary assessment (EMA). EMAs are brief surveys, often delivered daily or multiple times a day over a mobile or wearable device, that collect real-time information on an individual’s behaviors, as well as their internal (e.g., affective) and external (e.g., social setting) contexts. Self-monitoring one’s affective states in an intervention can enhance awareness of real-time feelings, and much like the example provided above, train a person to be more aware of their current affective state over time.

### PEAC and health equity

Offering individualized, affect- and socially-based interventions can also address health equity issues in a number of ways. Here, we define health equity as the situations in which everyone has a fair and just opportunity to achieve the best health outcome possible [see Centers for Disease Control and Prevention (2024)].

It is well-established that “one size fits all” interventions often fail to be effective for the entire population of people they are intended to benefit (Carpenter et al., 2020; Cohen et al., 2019; Pressman and Cross, 2018). For instance, not everyone who experiences an objectively traumatic event experiences it as traumatic or develops posttraumatic stress disorder (Bonanno et al., 2006). Such findings suggest these individuals are either not experiencing the same degree of negative affect in response to the event, or are able to rapidly regulate the negative affect they do experience without need for intervention support. It is also well-established that those who are economically advantaged and/or who have more social supports are more likely to seek care for their mental and physical health [see Healthy People 2030 (n.d.)], and so it is critical to identify how best to engage individuals who would otherwise avoid or disengage from such intervention programs.

Socio/environmental constraints should also be considered when designing and implementing interventions. For instance, regular religious participation or family gatherings may be sources of enjoyment around which interventions can be timed/triggered, or the intervention can be triggered to preemptively encourage participation in such activities to promote positive affect. Cultural context impacts perceptions and experiences of affective states and extant research suggests that high and low arousal emotions may be valued differently by individuals from distinct cultural backgrounds. Although the PEAC model suggests that the breakdown of engagement is particularly likely to occur at “extreme” levels of physiological arousal, what constitutes extreme may vary by sociodemographic context (e.g., race, ethnicity, income, age, cultural background). This represents an interesting and important empirical question and direction for inquiry. Future research should thus explore how to leverage affective and regulatory states to better engage individuals from a variety of populations.

### Privacy considerations for digitally delivered interventions

A challenge to the use of digitally delivered interventions are considerations around privacy, confidentiality, and safety (Carpenter et al., 2020). Privacy is particularly concerning when the intervention is collecting sensitive information (e.g., about illegal behaviors), and confidentiality can be at risk due to the geolocation and user activity data that is often captured by mobile and wearable devices. Ongoing advances in data security are improving the ability of researchers and other interventionists to encrypt data, password protect software and devices, and guide the development of plans to ensure identifying information is kept separate from data and only accessible to approved research or intervention team members.

### Relationship of PEAC to motivational theories

Self-Determination Theory (SDT; Deci and Ryan, 2012) and the Capability, Opportunity, and Motivation framework for Behavior (COM-B; Michie et al., 2011; West and Michie, 2020; Willmott et al., 2021) provide guidance on how to meet higher order needs or motivations that promote decisions and actions in line with behavior change. For instance, SDT suggests that the key psychological needs of autonomy, relatedness, and competence, which help increase self-efficacy and promote intrinsic motivation, must be met to achieve meaningful behavior change. COM-B suggests that the related components of capability, opportunity, and motivation are necessary for behavior change to occur. Similarly, the Motivation, Engagement, and Thriving in User Experience (METUX) model (Peters et al., 2018) provides a theoretically grounded framework to guide engagement at various points in a user’s experience interacting with a novel technology.

These existing models relate to the PEAC theoretical framework in interesting and important ways. The theorizing proposed in the PEAC model will facilitate, or in some cases even drive, several aspects of these existing theories. For instance, autonomy, which is central to SDT, relies on an individual feeling like an agent who is in control of making choices about and governing the outcomes of their life. In western cultures that highly value agency and autonomy, positive affect, a core component of the PEAC model, is associated with, interacts with, and/or promotes greater autonomy and agency (Bordbar, 2021; Chang et al., 2019; Deci and Ryan, 1987; Gentsch et al., 2015). Similarly, the affective processes outlined in PEAC are foundational to thriving and motivation (as per the METUX model). Thus, as stated above, we propose the PEAC model will guide the creation of the affective circumstances that promote engagement. This process will help initiate and sustain engagement even in moments when psychological needs may not be met, or when opportunities or motivations for engagement may be low.

How and to what extent existing motivational models may provide guidance, and interact with the PEAC model, to promote and sustain engagement over time, remains an empirical question. Future research should investigate the relationship between achieving the affective circumstances that promote and sustain engagement in behavior change, and the motivational factors described in these, and other complementary, behavior change theories.

## Discussion

The Precision Emotion and Affective Context (PEAC) Process Model of Engagement for Behavior Change proposed here aims to introduce a novel theoretical framework that guides predictions to further our understanding of how interventions can be structured to better promote and sustain engagement in positive behavior change. A major goal of this paper is to synthesize theories of affect and regulation, engagement, and motivation into a comprehensive model that highlights the processes underlying engagement in health and other positive behavior change interventions. We emphasize how use of the PEAC model can guide consideration of affective circumstances made by researchers and others delivering interventions, as well as the individuals themselves, to better promote engagement with various components of an intervention. This model can also guide those delivering interventions in identifying affective strategies (e.g., regulation techniques) that a patient can use to overcome barriers to engagement in behavior change interventions.

Our model can adapt to different levels of direct involvement with a client, patient, or participant. While we expect there would be some degree of human contact, at least initially, any method ranging from a person delivering an intervention with a direct hands-on approach, to a digital intervention that sends anonymous messages and responds based on algorithms (and any option in-between) could deliver and assess intervention-related content on an ongoing basis. The individual developing a connection with the interventionist or even the intervention itself is impactful (Carpenter et al., 2023a), which underscores the importance of assessing and addressing integral affect and adjusting the intervention accordingly. Thus, our model lays the foundation for an improved understanding of engagement in behavior change, which will plausibly also improve our ability to support individuals enrolled in interventions.

The literature reviewed here provides strong support for the PEAC model through examining the interplay of affective processes (valence, arousal), decision making, and behavior change. Although we do not present new data in the present paper, we lay the path for future studies where our proposed PEAC model can be tested empirically and further refined. We thus envision the PEAC model serving as a launching point or “call to action” to guide and encourage future affectively-driven research on engagement and its role in promoting and sustaining behavior change. We have provided an example of three different types of behavior change interventions, the first focused on the promotion of physical activity, the second on the promotion of engagement in a CBT intervention, and the third on the regulation of stress in a high-stress occupational context (among on-duty law enforcement officers). This theoretical framework will guide the construction of more efficacious and personalized interventions through providing testable predictions and highlighting key factors that must be considered when constructing the affective circumstances under which an intervention can most effectively promote engagement and subsequent positive behavior change.

## Data Availability

The original contributions presented in the study are included in the article, further inquiries can be directed to the corresponding author.
